# A new method for hosting and sharing MATLAB Web App

**DOI:** 10.1038/s41598-022-26165-3

**Published:** 2022-12-14

**Authors:** Ruijie Ren, Xiang-e Sun, Lin Hu

**Affiliations:** School of Electronic Information, Yangtze University, Jingzhou, 434100 China

**Keywords:** Software, Computer science, Electrical and electronic engineering

## Abstract

Currently, using MATLAB Web App Server to deploy MATLAB Web applications for hosting and sharing interactive Web applications involves the following problems: slow loading of applications, incompatibility with some browser versions, and various shortcomings of deployment tools, such as poor scalability and difficulty in optimizing black boxes. These problems adversely affect the user experience. To address this situation, we propose the use of front-end technology to design application layouts and build an application hosting platform with front-end and back-end separation using Nginx and Python. This method is shown to be simple and efficient, and it can successfully overcome the aforementioned problems while providing new ideas for hosting and sharing.

## Introduction

Although MATLAB is an excellent programming and numerical computation tool, it is difficult to run MATLAB applications outside the MATLAB environment, which is the main drawback from the users’ perspective. To address this problem, MATLAB launched MATLAB Web App in 2018. It can run MATLAB applications on the web browser side, with the aim of hosting and sharing user-defined MATLAB applications as interactive web applications on the cloud through the MATLAB Web App Server tool. To this end, users only need to enter the unique Uniform Resource Locator (URL) of the homepage of MATLAB Web App Server or the application itself in the browser, and it can be accessed and run outside the MATLAB environment without any configuration.

However, this method has the following shortcomings: slow loading of the application, poor compatibility of the browser, and single function of the servitization tool. This study analyzes the current development status and servitization of Web App based on MATLAB, discusses its compatibility, loading speed, application encapsulation, optimization, and other issues, and comprehensively examines the problem of slow loading speed. Finally, Python is selected to write the back-end hosted program in order to call the MATLAB application. The front-end Vue combined with Nginx can be used to realize visual deployment of the applications and thus overcome the aforementioned problems effectively.

## Related work

### MATLAB App Designer

The visual application design of MATLAB is usually achieved using GUIDE^1^ or MATLAB App Designer^2^. GUIDE is the main early development platform for users. It is based on Java Swing development with ActiveX control support, and it can be used to easily build a fully functional and modern application. However, Oracle is no longer investing in its development and its architecture is not conducive to Web workflows. Hence, MathWorks launched MATLAB App Designer as not only a new upgrade but also a replacement for GUIDE in 2016. MATLAB App Designer has a richer graphical user interface (GUI) visualization component and it provides a more stable way for programming app behavior compared to GUIDE. The HTML-based components significantly increase the scalability of the applications and facilitate the development of Web-based workflow services, while the new interactive programming approach allows writing of more reliable callback functions and sharing of data between different parts of the application.

Currently, MATLAB App Designer is widely used in the development of MATLAB Web App, which is packaged and compiled into Component Technology File (CTF) format using MATLAB Compiler and deployed on MATLAB Web App Server. Related work includes an electric vehicle performance simulation platform developed by N. Hinovet al.^3^ and an optical experiment virtual simulation system developed by YU Xinjie et al.^4^. All these studies have been conducted on the basis of App Designer and can be deployed to the cloud through the Web App server. However, there are some problems, such as compatibility and slow loading. Meanwhile, Bao Cong^5^, Yu Pan^6^, etc., investigated a combination of Java and MATLAB. They used JAVA interface programming technology and the WebFigure tag in JSP to realize the effect of calling the MATLAB GUI graphical form on the web side; however, the loading speed of the graphical form is extremely low. Li Jingyu^7^, Cui Peng^8^, etc. independently designed the dynamic link library for Simulink invocation and simulation. However, this method is not suitable for the black-box compilation of App Designer. Liu Y^9^ used the sh file to call the system compiler in order to compile the MATLAB file and present it on the web through Python. However, this approach is more about a MATLAB online solution.

### Current issues

At present, although the infrastructure for developing, hosting, and sharing based on MATLAB App Designer is well established, many problems persist, and they must be addressed.

#### Chromium 49 kernel browser is not supported

**Figure 1:**
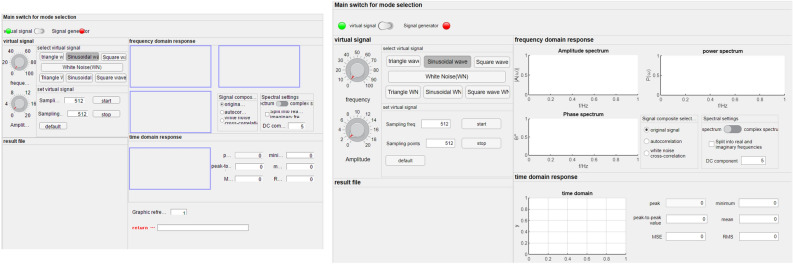
Loading applications on Chrome browser versions 49(left) and 99(right).

Chromium is an open-source browser kernel that has the largest market share, and it is used by nearly all major browsers (Microsoft Edge, Opera, etc.). In our tests, we found that many apps are not fully functional or they do not display complete information for Chromium versions below 49. In general, applications with MATLAB 2020a suffer from missing components, whereas applications with versions 2020b and above do not display properly. In many countries, owing to the accumulation of historical problems, the equipment update iteration is extremely slow. In the multi-point office environment based on MATLAB, Chromium49 is the highest version of the Chrome browser supported by the Windows XP system, and its incompatibility will become the main obstacle to the project. As shown in Fig. 1, the virtual signal simulation application of MATLAB 2020a is considered as an example. The blue box part is not fully displayed in the browser test on Chrome 49 and Chrome 99.

#### MATLAB Web App loads slowly on the browser

We found that applications of MATLAB Web App load extremely slowly after deployment. The average loading time is around 12–16 s on some old computers and around 6–8 s on some new models. In particular, systems with Intel processors are generally slower than those with AMD processors.

Here, a loading test is conducted for blank components, single components, and apps composed of multiple components in MATLAB App Designer. Among them, ctf is the file format supported when the application is service-oriented, and mlapp is the file packaged by the app designer that can be run locally. When different components are loaded, the number of requests sent for the first time is roughly the same, and the first run time is relatively long. The deployment data obtained after several rounds of control loading tests and comparisons are summarized in Table 1 (Detailed data can be obtained from supplementary information).

**Table 1:** Comparison of loading times of different components.

App_name	App_size	ctf_size	First load request	First run time	First refresh time	mlapp run time
Blankcomponent	7 kb	1312 kb	53	2.39 s	2.33 s	1.17 s
Radio list box	10 kb	1312 kb	53	3.52 s	2.09 s	2.60 s
Slider	10 kb	1312 kb	54	3.32 s	2.74 s	2.77 s
Table	9 kb	1312 kb	53	3.79 s	2.08 s	3.24 s
Calculator	25 kb	1323 kb	54	3.52 s	2.12 s	1.97 s
FlightInstruments	101 kb	1741 kb	55	7.86 s	2.73 s	3.83 s
Virt_Signal_Simulation	234 kb	1463 kb	55	8.62 s	3.07 s	4.41 s

#### Closed-source packaging and compilation of applications makes it difficult to study their kernel functionality and optimize them

The MATLAB Compiler tool compiles the code in the M-file on a C/C +  + interface and executes it by calling the MATLAB Compiler Runtime (MCR) runtime library component resource. This process is encrypted using an Advanced Encryption Standard (AES) key, and the resulting CTF compresses the archived file to secure the application source code, making it difficult for users to explore the internal implementation principle and optimize it.

#### Custom component process is complex

Previously, users used the java-component function on the GUIDE platform to add custom components and enrich the interactive functionality with undocumented java-frame properties. This process requires engineers to have a high level of experience and technical skills. Currently, users configure the metadata of the App Designer tool by specifying the UI component classes for a class file on the App Designer platform. However, the complex design aspects and limited operational space restrict the user's ability to extend the functionality.

#### Deployment tools have poor scalability, security risks, and various deficiencies

MATLAB Web App Server uses industry-standard authentication and access control protocols to protect users' Web applications and data. In addition, it provides support for cross-version development of Web applications for most application scenarios. However, it does not provide an external extension interface, and some complex scenarios such as the need to divide the deployment zone according to specific people in the physically isolated network space cannot be implemented directly. Meanwhile, it is recommended that the server be run only in a trusted intranet environment and not be open to the extranet; otherwise, it would be prone to risks such as code injection. Finally, the tool has many deficiencies, such as the possibility of mutual interference when users and programs access each other at the same time. In addition, the server restart process is often accompanied by a service error message, typically “NET HELPMSG 2244”, which can be resolved by removing redundant user registration information. In terms of server compatibility with multiple versions of Runtime, the server tends to work only on the first MATLAB Runtime path in the configuration list.

### Research on application packaging and compilation mechanism

The applications supported by MATLAB Compiler^10^ mainly include independent applications, C/C +  + shared libraries, Excel plug-ins, and various types of .COM and .NET objects. Their dependencies are analyzed in turn, and code is generated with C/C +  + interfaces. Subsequently, the code is encrypted using AES and an archived CFT file is created. Finally, the aforementioned processes are closed for execution. During this process, the CFT file will extract resources in the MCR, decompress them (keys, etc.), and generate some M files related to DICOM reading and writing in the toolbox folder, which are presented in an unintelligible form in MATLAB Editor. It is extremely difficult to improve the application loading speed from this perspective.

### Research on MATLAB Web App slow loading problem

We found that the following are the likely factors that affect the loading speed of MATLAB Web App: the invalid overloading of the MCR runtime components, the forced updating of static resources by the server, and the lack of a session management function of the web server.

#### MCR runtime library components

In our tests, we found that single-function applications made a similar number of requests, while complex applications required additional requests because they needed to load additional MCR component resources. By preloading the commonly used component resources to the browser in advance, one can avoid the invalid reloading of the same component resources, and the application loading speed will be improved considerably. However, the wrapper in App Designer is a black box that does not allow one to explore how it compiles, which makes it difficult to optimize the loading of the runtime components.

#### Static cache resources

In the statistics of the load time of the application components, we found that for the same application, when the page is refreshed within a short period of time, the load time of the application is shorter, and when the page is refreshed after a certain period of time, the load time of the application is restored to the same as that of the first load. This is because for a short time, the browser caches the static resources of the application in order to facilitate the second access, and MATLAB Web App Server defaults to update the static resources; after a certain period of time, these static resources need to be re-requested. By consulting numerous official documents under the installation path of MATLAB Runtime, one can limit some cache updates by modifying the configuration file named webapps.config. However, the improvement of this method is very limited.

#### Session manager

In Web App service requests, we found that the server took a long time to respond to session requests from the browser, and in most cases, reloading the application updated the sessionID. Specifically, when the application is loaded for the first time and refreshed within a short period of time, it initiates a command request to establish a newSession, which takes a long time to respond to the session. When the application interface is refreshed after a certain period of time, the command request is displayed as trackSession, indicating that the session is still maintained. This indicates that MATLAB Web App Server does not have session management capabilities. This is also confirmed by looking at the server process ctfxlancher.exe. We use cross-domain technology to build a session management system based on Spring Boot + Vue, and we store the session information in the local database so that the application can use the original parameters to connect to the server background and achieve a speed-up effect.

**Figure 2:**
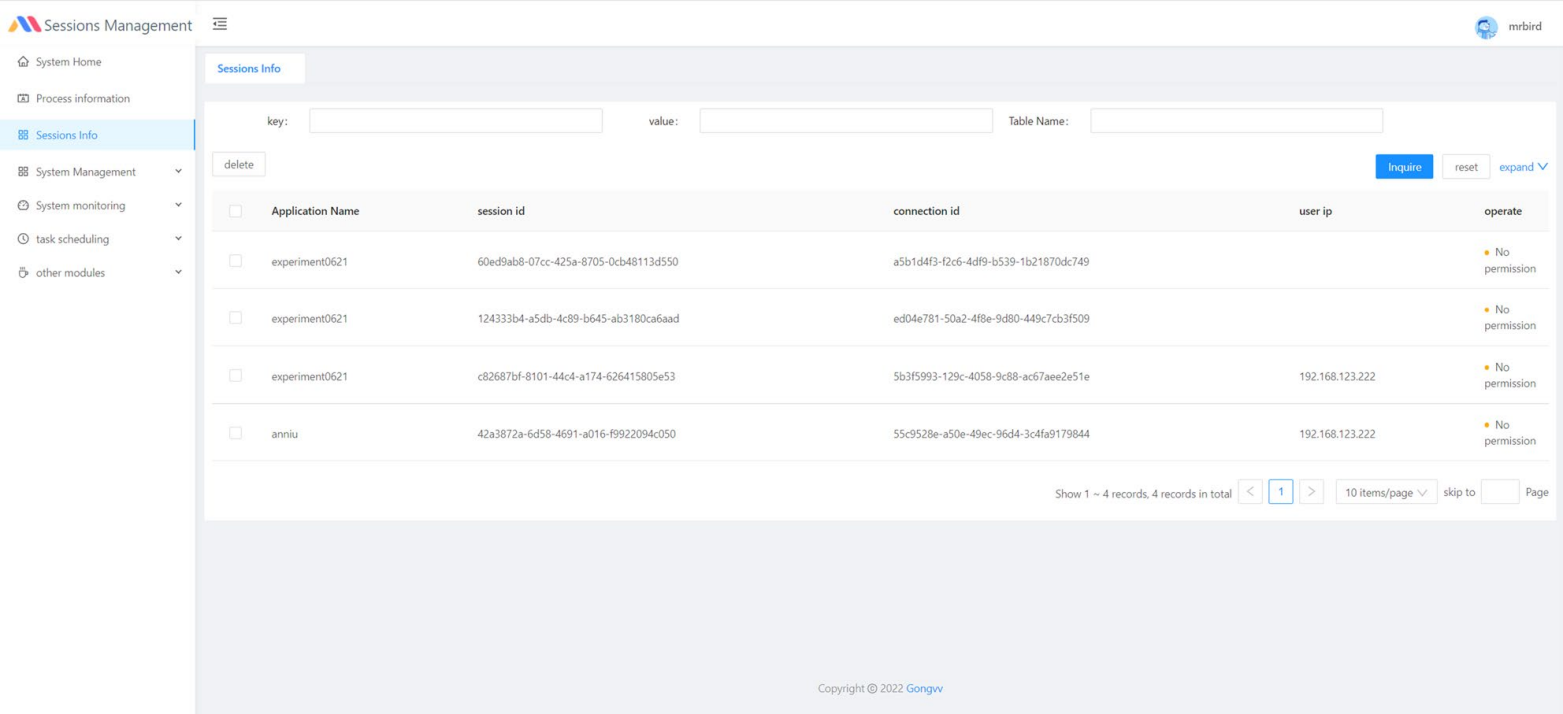
Session information in the session manager.

**Figure 3:**
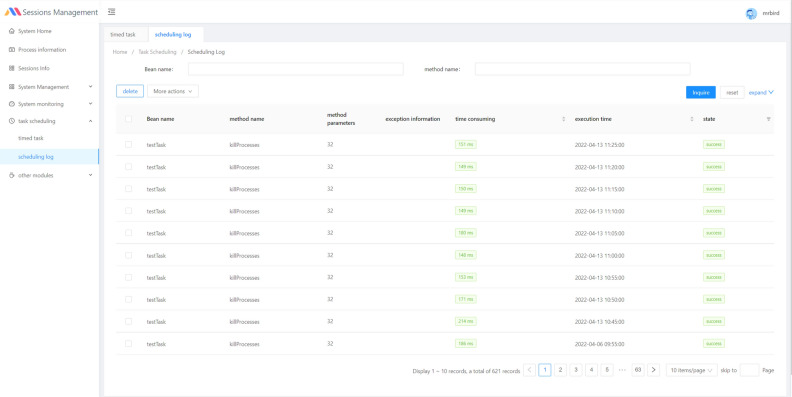
Process management log interface in the session manager.

Pull the mirror image of Mysql, Redis, and Nginx on Docker, and complete the deployment of the front and back ends. The session information management page of the session management system is shown in Fig. 2, it contains the session id, connection id, and user information stored by the user. The scheduled task management log is shown in Fig. 3. The parameter of the scheduled end process is 32, which is the maximum number of processes allowed by the server, and the execution rule of process management is the crontab expression. This method can effectively improve the loading efficiency of the application; however, it is not flexible in the long run.

### Investigating previous methods for implementing similar App Designer functions

Considering the issue of optimizing the loading speed of MATLAB Web App, we actively explored technologies and methods that can implement App-Designer-like functions, in addition to identifying breakthroughs in the MATLAB application itself.

#### Modelit Webservice Toolbox for MATLAB

The Modelit toolkit is used for deploying MATLAB code as a Web service in a simple manner and at the lowest possible cost. Its core is a servlet that redirects incoming Web requests to MATLAB callback functions, enabling tasks such as encapsulating MATLAB graphics in HTML, converting JSON strings to MATLAB, and parallelizing Web services through asynchronous calls. In practice, although the toolkit used to create Web services implementing GUI callbacks have a great advantage in terms of the page loading efficiency, the complex configuration of the environment and the lack of official follow-up support are serious obstacles.

#### Octave Web-interface

The Octave Web interface^11^ is a browser interface for the open-source MATLAB clone GNU Octave (in combination with GNU plot). It has the characteristics of high compatibility with MATLAB programs, small size, and a rich community. We tried to mount the MATLAB Web App to its service and found that the application could not implement Simulink functionality; the code was particularly inefficient. In addition, Octave has no alternative to App Designer or GUI compared to MATLAB, and the ability to create standalone applications is limited.

### Existing related technology research

#### Can MATLAB be combined with front-end and back-end architectures?

MathWorks provides users with various means to run MATLAB products in the cloud, such as using MATLAB Online to run MATLAB and Simulink applications online, accessing parallel computing clusters in Amazon Web Services (AWS) through MathWorks Cloud Center, and building Docker container images to deploy MATLAB in cloud and server environments. These cases comprehensively demonstrate the feasibility of combining MATLAB with front-end and back-end architectures.

Furthermore, MATLAB deployment tools provide REST APIs for synchronously or asynchronously executing application functions as well as for monitoring the server operation status, which are fully consistent with the current mainstream Web architecture style. In addition, MATLAB can be combined with front-end and back-end architectures using Ajax to handle asynchronous requests, separating the front-end and back-end, and optimizing the server performance through API specification design^12^.

#### Research on front-end implementation methods

A low-code platform^13^ is one of the most intriguing technologies in the current science and technology industry. It can rapidly realize digital applications by means of graphics and drag-and-drop, thereby reducing the threshold of application development considerably. Moreover, it has equally satisfactory results compared to App Designer. To a certain extent, we believe that it can meet most functions of App Designer. To this end, we investigated more than 200 mainstream low-code platforms (Obtained in supplementary information) in the current market and selected Vue-Layout, Luban H5, and web_designer for development according to whether the code is open source, whether it can customize the components, and whether it can add requests and responses to the controls. However, in practice, nearly all these platforms have problems such as unstable service and incomplete function, and their creators are independent developers; hence, it is difficult to guarantee follow-up maintenance of the platforms.

Compared with the lack of a low-code platform, it is advisable to directly use a mature front-end UI framework and Vue to rapidly build a fully functional application view. This method has a high degree of freedom. The project can add user management, cache optimization, exception handling, and other functions according to the requirements.

#### Research on back-end implementation methods

MATLAB Production Server is an official enterprise production application that integrates MATLAB applications into Web and database services. It runs on a dedicated server or cloud, and it has most of the functions of MATLAB Web App Server with stronger data integration and features such as management and monitoring. However, it also has some disadvantages, i.e., it is expensive and incompatible with MATLAB Web applications, e.g., it is unable to interactively run the applications intuitively.

As MATLAB itself provides external language interfaces^14^ such as C/C +, Java, Fortran, and Python, it supports mixed programming among multiple languages. Hence, it is possible to build a MATLAB application hosting platform with external languages. Considering the simplicity and efficiency of Python, and as some Web frameworks such as FastAPI have extremely high performance comparable to Go and NodeJS, it is more practical to select Python^15^ to build the back-end server.

#### Final program determination

Considering the factors discussed above, we finally selected FastAPI as the back-end framework to write the application hosting application. Further, we used the Vue front-end framework to create a REST API to connect with the back-end, and we combined it with the Nginx server to complete the sharing. This method is simple and efficient, avoids the problem of MATLAB black-box encapsulation, and has a mature framework and rich community resources that provide effective support for the continuous development of the project.

## Method

### Overall architecture design

**Figure 4:**
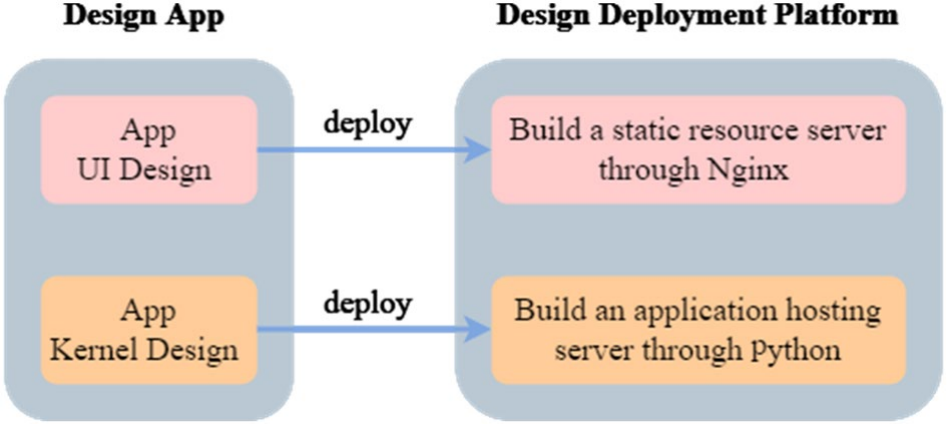
Application design introduction.

This method divides the Web App design into two parts, i.e., the application UI design and the application kernel program design, and it combines Python with Nginx^16^ to build a server deployment platform with front-end and back-end separation instead of MATLAB Web App Server. As shown in Fig. 4, the static resources of the application are deployed on the Nginx server and the kernel program resources of the application are deployed on the back-end Asynchronous Server Gateway Interface (ASGI) server.

**Figure 5:**
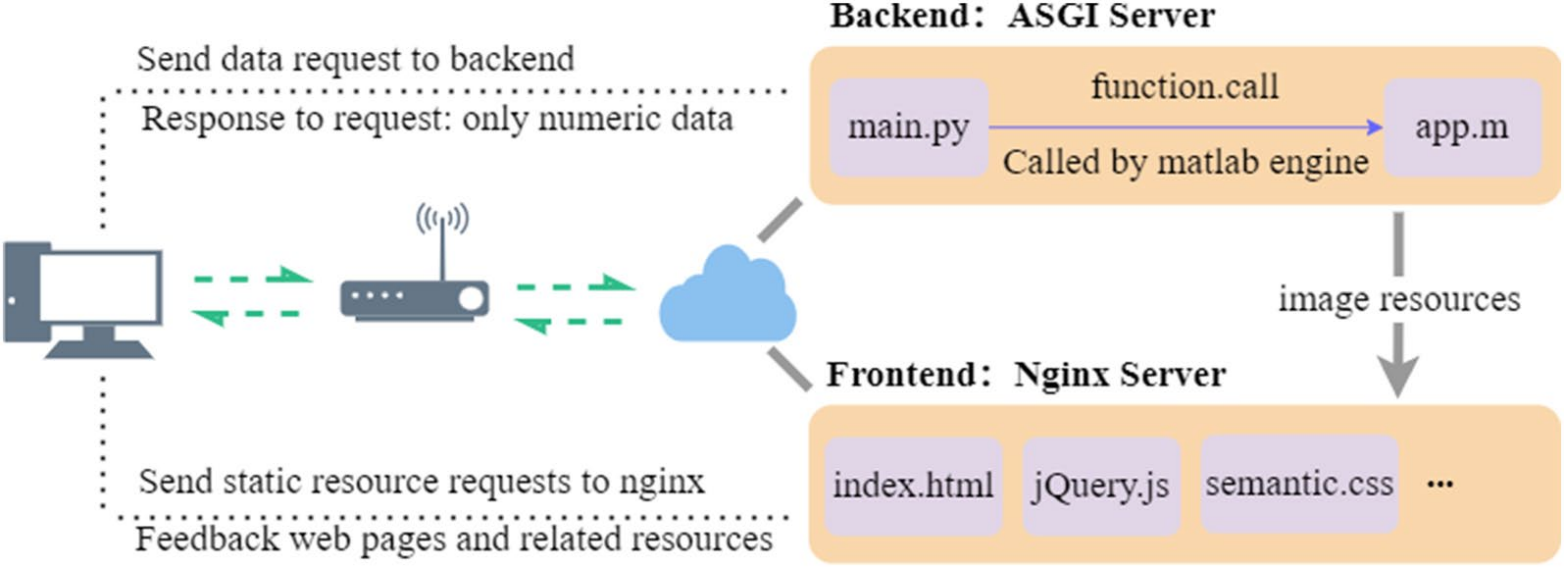
General architecture of MATLAB Web App Server replacement solution.

The overall structure of MATLAB Web App hosting and sharing is shown in Fig. 5. The user enters the unique URL of the application home page or the application itself on the browser side. The relevant static resources such as html, js, css, and default icons can be obtained through the Nginx server, and they are finally displayed on the user's screen. When an error occurs on the front-end server, the user can obtain the html resource containing the error message and determine the cause of the error. The user operates and sends a REST request to the back-end to connect with the back-end API. The back-end main.py program calls the MATLAB program in the same directory for calculation and directly feeds back the numerical results to the browser, while other results such as images are first transmitted to the Nginx server and then fed back to the user.

### Design of Web App

In this study, we consider the application of filters in digital signal processing, which is a core subject for telecommunications students^17^, as an example. The development environment comprises Windows 11, Nginx 1.20.2, Python 3.6.13, and MATLAB 2020a. The test environment is macOS Monterey 12.3, and the browser version is Chrome 99.

For the UI design of the application, the Semantic UI framework is chosen to create the application layout, jQuery^18^ is chosen as the JavaScript library to write the application response function, and the front-end framework Vue is finally used to create the REST API to connect with the back-end, with the following key code. The address communicated with the back end is the IPv4 address of the local machine, which can be obtained by entering ipconfig in the terminal. Here, res receives the calculation result from the back end and assigns it to getdata, where getdata represents the minimum order of the filter.
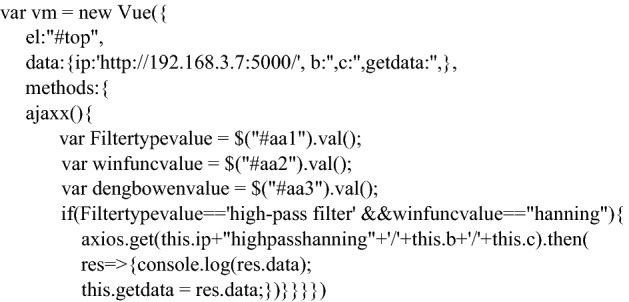


The kernel program for the application is written in MATLAB. The storage path for the generated images is set to the Nginx static resource root directory.

### Deployment of Web App

The Web App deployment consists of two parts. The first part is the deployment of application UI resources, i.e., the application home page index.html, static resources css and js, application icons favicon.ico, etc., which is achieved by the static resource server Nginx. The second part is the deployment of the application kernel program through the FastAPI^19^ framework to achieve the front-end and back-end REST API interface.

Create a new static resource server on Nginx and add the UI resources of the Web App into it. Locate the configuration file nginx.conf in the directory named conf, add the server path, and set the index to point to the target html. Further, add the monitoring domain name and port number, and start the Nginx server in the terminal. It should be noted that the MATLAB program must be placed in the directory at the same level as the back-end Python program. Although this operation is not necessary and can be performed by setting the path, path positioning failure may occur frequently. Finally, set the back-end server gateway to the local IPv4 address. Start the ASGI server to complete the deployment.

The key code of part of the back-end is as follows. Here, highpasshanning in the routing path in the code is the functional interface name of the application, where b and c are the two parameters required to implement the function. Further, eng is an instantiation of MATLAB. In the case of hosting multiple versions of MATLAB applications, one needs to use sys.path.append to add the engine package to the environment variables. Uvicorn is a lightweight web services framework with the same address and interface as the front end.
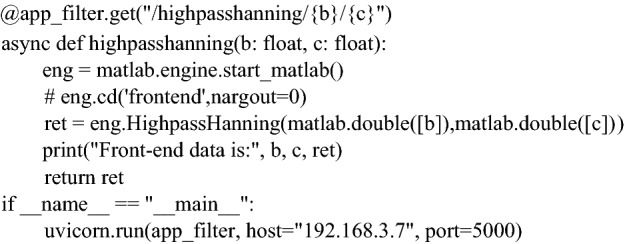


## Experiment

**Figure 6:**
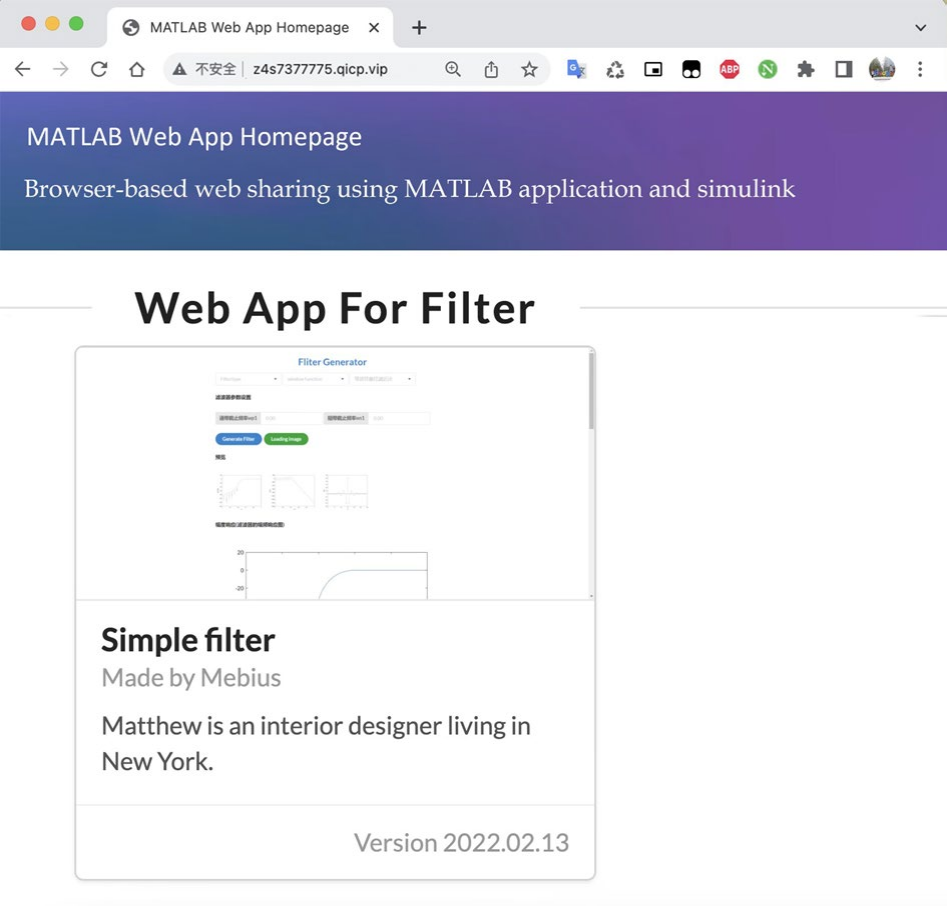
Web implementation of the application homepage.

After the server is simply mapped using the Network Address Translation (NAT) tool, the user only needs to enter the homepage domain name address or the unique URL of the target application in the browser to access the interface page of the filter application. We ran tests on different versions of Chrome and browsers with different kernels, such as Safari and Firefox, and all the tests were normal. In the case of Chrome 99, the homepage of the application is shown in Fig. 6.

**Figure 7:**
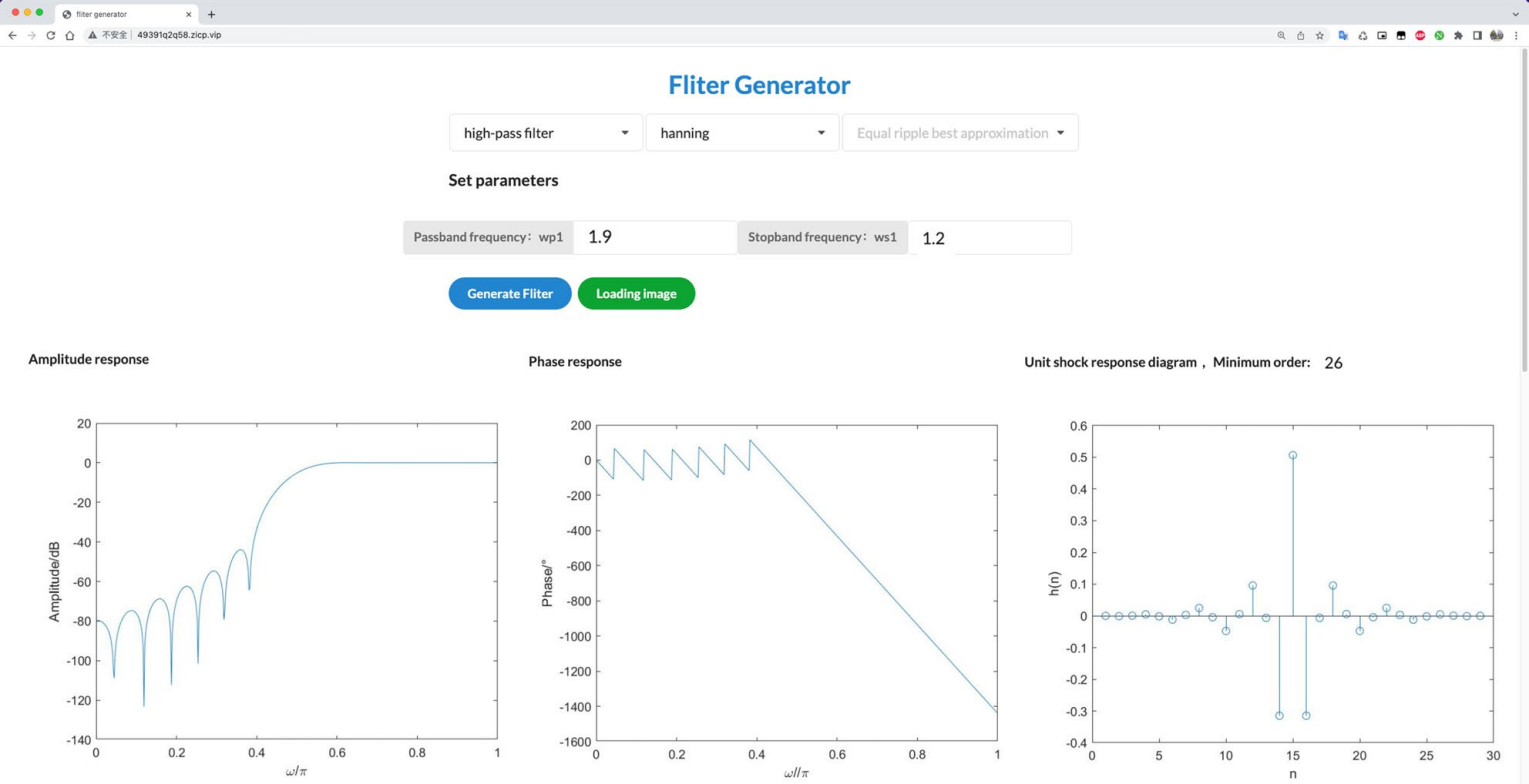
Web-side implementation of the filter application.

Click the Simple Filter application to enter the application interface page, as shown in Fig. 7. Here, select the filter as a high-pass Hanning window, enter the relevant parameters, and click the submit button to obtain the cloud feedback results. The minimum order of the current filter is 29, and the amplitude frequency, phase frequency, and unit shock response plots are consistent with the results in MATLAB. Once the application is deployed, it can be tested using the interactive API documentation generated from the built-in Swagger UI.

Thanks to the fast loading speed of static web pages, in the experiment, we can access the application interface almost without waiting. At the same time, we can use css to decorate the application interface and enrich the expression of the application. In terms of personalized needs, the nature of the open source framework gives us more options to create, optimize, and maintain new functionality for applications.

## Conclusion

This paper presented a new method for MATLAB Web App hosting and sharing. In this method, the application visualization resources built using front-end technology are stored on the Nginx server, and the back-end MATLAB programs are deployed in the hosted and shared server written in Python. The two ends communicate through RESTful API. This method significantly improves the tension and smoothness of the application interface while allowing the application to open within a few milliseconds. Compared to traditional methods, the experience is superior, It comprehensively solves all the problems existing in current traditional methods. For the Modelit toolbox and low-code platforms, one need not worry about complex environment configurations and whether the platform can be maintained later. Our approach is functional and efficient for the Octave Web interface, with no porting costs. In addition, this method is not subject to the influence of MATLAB black-box packaging, and it can reasonably control the release of static cache and process session management to overcome the problems of traditional compilation, such as difficulty in optimization and slow loading of the servitization application.

However, this method has some shortcomings. For example, images and other resources in the back-end response data can be stored in a cache database such as Redis so as to avoid confusion in the case of data returned multiple times. When multiple Vue Routers point to the same component, the page will occasionally fail to refresh; hence, the reused component must be monitored. In the multi-point local office environment, when the concurrent ability of services is not sufficiently prominent, we can consider using Go language to structure the service programs. Future work will focus on improving and perfecting the system function so as to meet a wider range of user needs (Supplementary Information).

## Supplementary Information

Supplementary Information.

## Data Availability

The datasets generated and analyzed during the current study are available in the github repository, [https://github.com/mebius95/A-New-Method-for-Hosting-and-Sharing-MATLAB-Web-App].
